# The Functions of TRIM56 in Antiviral Innate Immunity and Tumorigenesis

**DOI:** 10.3390/ijms24055046

**Published:** 2023-03-06

**Authors:** Lin Fu, Xiaotong Zhou, Qian Jiao, Xi Chen

**Affiliations:** School of Basic Medicine, Qingdao University, Qingdao 266000, China

**Keywords:** TRIM56, innate immune response, antivirus, tumor

## Abstract

As a member of the TRIM (tripartite motif) protein family, TRIM56 can function as an E3 ubiquitin ligase. In addition, TRIM56 has been shown to possess deubiquitinase activity and the ability to bind RNA. This adds to the complexity of the regulatory mechanism of TRIM56. TRIM56 was initially found to be able to regulate the innate immune response. In recent years, its role in direct antiviral and tumor development has also attracted the interest of researchers, but there is no systematic review on TRIM56. Here, we first summarize the structural features and expression of TRIM56. Then, we review the functions of TRIM56 in TLR and cGAS-STING pathways of innate immune response, the mechanisms and structural specificity of TRIM56 against different types of viruses, and the dual roles of TRIM56 in tumorigenesis. Finally, we discuss the future research directions regarding TRIM56.

## 1. Introduction

The tripartite-motif (TRIM) family of proteins, also known as really interesting new gene (RING)-B-box-Coiled-Coil (RBCC) region proteins, is composed of an N-terminal RING structural domain, one or two B-box patterns, and an α-helical coiled-coil domain, followed by a highly variable carboxyl structural domain from the N-terminus to the C-terminus [1,2,3]. TRIMs are a large family of proteins, and approximately 80 members of the TRIM family have been identified in humans [4,5]. Based on the highly variable C-terminal structural domains, TRIMs with RING structural domains can be classified into subfamilies I to XI (C-I to C-XI). The variable C-terminal regions include the PRY structural domain, the SPRY structural domain, the COS structural domain, the fibronectin type III repeat region (FNIII), the acid-rich region (ACID), the Meprin and TRAF-homologous structural domain (MATH), the ADP-ribosylation factor family structural domain (ARF), the filamine-type IG structural domain (FIL), the NHL structural domain, the PHD structural domain, bromodomain (BROMO), and the transmembrane region (TM) [3,6,7].

TRIM family members are involved in a wide range of cellular activities and biological processes, including DNA damage repair [8], RNA binding [9], autophagy [1,10], apoptosis [11], cell cycle [12], viral infection [13,14], immune activation [7,15], inflammatory processes [16], stem cell differentiation [17], and neurogenesis [18,19]. The aberrant expression of TRIM family members leads to the development of various diseases, including tumors and neurological disorders [19,20,21].

TRIM56 is a member of the TRIM family. TRIM56 was originally reported to regulate the intracellular double-stranded DNA innate immune response [22]. In recent years, an increasing number of studies have shown that TRIM56 is involved in the host response to viral infection. On one hand, TRIM56 acts by regulating host innate immune signaling. TRIM56 is able to cause the transcriptional induction of pro-inflammatory cytokines and type I interferon (IFN) by regulating the toll-like receptor (TLR) signaling pathway and the cyclic GMP-AMP synthase (cGAS)-stimulator interferon gene (STING) signaling pathway to limit viral transmission [23,24,25,26,27]. On the other hand, as a direct antiviral restriction factor, TRIM56 has been shown to have a direct antiviral effect on positive single-stranded RNA viruses of the Flaviviridae, Coronaviridae, and Retroviridae families. In addition, it is also effective against negative single-stranded RNA viruses (influenza A and B) and two DNA viruses [28].

The expression level of TRIM56 is not consistent among different tumor types, and its expression changes are closely related to tumor development and prognosis. This suggests that TRIM56 may play different pro- or anti-cancer functions in different tumor types. In recent years, many studies have revealed the function of TRIM56 in tumor development. TRIM56 is an oncogene in glioma, breast cancer, and Kaposi’s sarcoma [29,30,31,32], but it is a tumor suppressor in ovarian cancer, multiple myeloma, lung adenocarcinoma, hepatocellular carcinoma, and leukemia [33,34,35,36,37].

Here, we first describe the structural features and expression characteristics of TRIM56. Next, we focus on reviewing the role of TRIM56 in innate immunity and antiviral processes. We also summarize the role of TRIM56 in tumors. Finally, we discuss the future directions of TRIM56 research. Reviewing the antiviral and tumor regulatory functions and specific mechanisms of TRIM56 is beneficial to provide new ideas for developing novel antiviral drugs and enriching therapeutic strategies against tumors.

## 2. Structure and Expression of TRIM56

**Figure 1:**
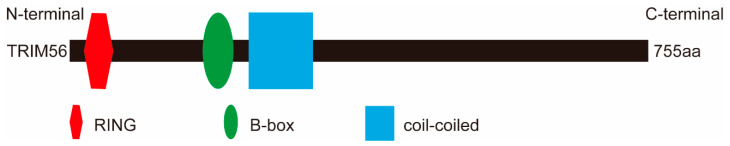
Schematic representation of the domains of TRIM56. TRIM56 has three structural domains, an N-terminal RING domain (red), a B-box domain (green), and a coiled-coil domain (blue). The human TRIM56 transcript is 755 aa long.

TRIM56, also known as Ring finger protein 109 (RNF109), is an 81 kDa protein of 755 amino acids encoded by the TRIM56 gene on human chromosome 7. The protein contains three structural domains, a RING domain, a B-box domain, and a coiled-coil domain (Figure 1). Because it lacks a C-terminal structural domain, TRIM56 belongs to the C-V subfamily.

The RING structural domain is a unique linear sequence of cysteine and histidine residues in a zinc finger structural domain that forms the catalytic center of the ubiquitinating enzyme. Ubiquitination is a very important post-translational modification process that plays roles in innate immune and tumorigenic development [38,39,40]. Ubiquitin is a 76-residue polypeptide. The key enzymes required for the ubiquitination process are ubiquitin-activating enzyme E1, ubiquitin-binding enzyme E2, and ubiquitin-ligase E3 [41]. Among them, E3 ubiquitin ligase can catalyze the covalent binding of ubiquitin molecules to substrates. E3 ubiquitin ligases can be classified into several groups according to their specific structural domains: the RING family, the family of homologous to E6AP C-terminus (HECT), RBR E3s, and those of unclassified type [42]. As we mention above, most members of the TRIM family have a RING structural domain, and most TRIM members have been identified as functional E3 ubiquitin ligases [43]. Notably, a few TRIM proteins do not contain any RING domain, such as TRIM14 and TRIM66 [6].

The B-box structural domain consists of small peptide sequences that contain finger-like protrusion. Although the B-box structural domain also contains a “zinc finger” structure, it generally does not exert E3 ubiquitin ligase activity. There are two distinct isoforms of the B-box, B-box1 and B-box2. Most TRIM proteins contain one B-box2 structural domain or two B-box structural domains, while a few TRIMs, such as TRIM69, do not have either structural domain [44]. The B-box structural domain is thought to be involved in the recognition of target proteins by TRIM proteins [4]. The coiled-coil structure domain of TRIMs can serve as a scaffold for mediating the homomeric and heteromeric assembly of TRIMs and other proteins. Additionally, it also exhibits enzymatic or nucleic acid binding activity [21,45].

TRIM56 can act as an E3 ubiquitin ligase that catalyzes the ubiquitination of Vimentin, DVL2 (Dishevelled-2), ERα, SAP18 (Sin3A associated protein 18), IκBα, STING, cGAS, and TGF-β-activated kinase 1 (TAK1) [23,31,32,33,36]. Interestingly, TRIM56 also has deubiquitinating enzyme activity and the ability to bind RNA [29,30,46]. The relationship between the exertion of these functions and the structure needs to be further investigated.

TRIM56 is widely expressed in various tissues of adult mammals [47]. Similar to many other TRIM proteins, the expression of TRIM56 is regulated by type I IFN. The expression level of TRIM56 was significantly upregulated in cells after type I IFN treatment [22,48]. There are differences in the subcellular distribution of TRIM proteins [49]. Some TRIM proteins are widely distributed in the cytoplasm and nucleus, such as TRIM30 and TRIM32. Some are only present in the nucleus, such as TRIM19, and some are only present in the cytoplasm, such as TRIM29. In resting cells, the TRIM56 protein is only present in the cytoplasm and thus interacts with cytoplasmic proteins [47].

## 3. Antiviral Effects of TRIM56

TRIM family members have direct and indirect antiviral effects, including direct interactions with virus-associated proteins or nucleic acids, or modulation of antiviral signaling pathways associated with host immune function [15]. In recent years, numerous studies have demonstrated that TRIM56 exerts antiviral effects. TRIM56 can affect viral replication by modulating signaling pathways of the innate immune response. In addition, TRIM56 can directly target viral components to affect viral replication or inhibit their function, thereby exerting antiviral effects. Here, we summarize the studies on the interaction between the TRIM56 protein and viruses, in particular the role of TRIM56 in the signaling of the innate immune response and the direct interaction between TRIM56 and viruses.

### 3.1. TRIM56 Regulates the Antiviral Innate Immune Response

**Figure 2:**
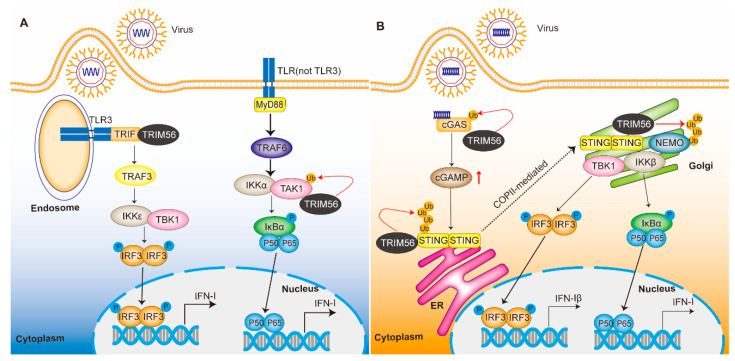
Regulatory network of TRIM56 in innate immunity. (**A**) TRIM56 in the TLR signaling pathway. TRIM56 catalyzes the M1-type ubiquitination modification of TAK1 and thus the interaction between TAK1 and IKKα. TRIM56 interacts with TRIF to positively regulate the TLR3-mediated interferon pathway in an E3-independent manner. (**B**) TRIM56 in the cGAS-STING signaling pathway. TRIM56 induces the Lys335 monoubiquitination of cGAS, resulting in a significant increase in cGAMP production. TRIM56 catalyzes the formation of the K63-linked ubiquitination of STING. This modification induces STING dimerization, which recruits TBK1 and induces IFN-1β. TRIM56 synthesizes a ubiquitin chain that binds to NEMO and mediates the ubiquitination of NEMO to activate IKKβ. P, phosphate; Ub, ubiquitin.

Innate immunity is the first line of defense against pathogen invasion. Upon pathogen invasion, pathogen-associated molecular patterns (PAMPs) are recognized by the pattern recognition receptors (PRRs) of innate immune cells, including retinoic-acid inducible gene-I (RIG-I)-like receptors (RLRs), TLRs, and cell membrane DNA receptors [50,51]. The triggering of PRRs ultimately leads to the activation of various signaling pathways and the transcriptional induction of pro-inflammatory cytokines and type I IFN to limit viral transmission [52,53]. Type I and type III IFNs are potent antiviral agents. They efficiently induce the production of hundreds of interferon-stimulated genes (ISGs) via the JAK-STAT signaling pathway, establishing an antiviral state by controlling and limiting viral infection and replication [54]. By modulating the innate immune response signaling pathway, TRIM56 can regulate downstream interferons and ISGs to precisely exert antiviral immune responses (Figure 2).

#### 3.1.1. Regulation of TLR Signaling Pathway

TLRs are the first-known PRRs capable of recognizing extracellular viral components that enter the cytoplasm by phagocytosis or endocytosis to induce type I IFN (IFN-I) and pro-inflammatory cytokines to counteract viral invasion. The TLR family contains 13 members [55]. Upon activation, all TLRs, except for TLR3, recruit adaptor molecule myeloid differentiation factor 88 (MyD88), which recruits kinase IL-1 receptor-associated kinase 1/4 (IRAK1/4) and E3 ubiquitin ligase TNF receptor-associated factor 6 (TRAF6). TRAF6 catalyzes its own ubiquitination. Ubiquitinated TRAF6 recognizes TAK1/MAP3K7 binding protein 2 (TAB2) and activates TAK1, ultimately leading to the activation of IκB kinase α/β/γ (IKKα/β/γ) and NF-κB [56,57].

TRIM56 catalyzes the M1-type ubiquitination modification of TAK1, which enhances the interaction of the TAK1-IKKα complex. The overexpression of TRIM56 enhances the TNF-α-induced activation of NF-κB signaling, whereas the knockdown of TRIM56 has the opposite effect. The C-terminus is the binding region of TRIM56 to TAK1, while the RING structural domain of TRIM56 is the active region of the E3 enzyme and is important for the ubiquitination of TAK1 [23].

Unlike other TLRs, TLR3 uses adaptor Toll-IL-1 receptor (TIR) domain-containing adaptor inducing IFN-β (TRIF) and then activates IRF3 via TBK1/IKKε-mediated phosphorylation. Phosphorylated IRF3 forms a dimer and then translocate to the nucleus, initiating IFN-I expression [58]. TRIM56 was found to interact with TRIF, which positively regulates the TLR3-mediated interferon pathway. This mechanism is independent of E3 ligase activity. The deletion of the C-terminus of TRIM56 abolished TRIM56-TRIF interaction and the enhancement of the TLR3-mediated IFN response [24] (Figure 2A). Furthermore, the overexpression of TRIM56 inhibits PEDV replication by positively regulating the TLR3-mediated antiviral signaling pathway [59].

#### 3.1.2. Regulation of cGAS-STING Signaling Pathway

cGAS, also known as MB21D1/C6orf150, is considered to be one of the most important cell membrane DNA sensors [60]. Upon recognition of viral DNA, cGAS synthesizes a second messenger molecule, cyclic GMP-AMP (cGAMP), which binds and activates STING and the transfer of STING from the endoplasmic reticulum (ER) to the Golgi apparatus via COPII-mediated vesicles [61]. Activated STING recruits and activates TBK1 and IKKβ, which promote the nuclear import of IRF3 and NF-κB, respectively, ultimately producing IFN-I and pro-inflammatory cytokines [62,63]. TRIM56 induces the Lys335 monoubiquitination of cGAS, resulting in a marked increase in cGAMP production, STING dimerization, and DNA-binding activity. TRIM56-deficient cells are defective in cGAS-mediated IFNα/β production during herpes simplex virus-1 (HSV-1) infection [25].

Tsuchida et al. found that the overexpression of TRIM56 enhanced IFN-β promoter activation after double-stranded DNA stimulation. TRIM56 interacts with STING and uses it as a substrate for lysine 63-linked ubiquitination. This modification induces STING dimerization, which recruits antiviral kinase TBK1 and induces IFN-β [22]. Ubiquitin regulatory X domain-containing proteins 3B (UBXN3B) can positively regulate STING signaling. Sting-/- mice and Ubxn3b-/- mice are highly susceptible to lethal HSV-1 and vesicular stomatitis virus (VSV) infections. UBXN3B interacts with STING and its E3 ligand TRIM56 and promotes STING ubiquitination, dimerization, translocation, and the subsequent recruitment and phosphorylation of TBK1 in the innate immune processes [26]. However, recent studies have shown that TRIM56 does not directly ubiquitinate STING. Wang et al. found that TRIM56 cannot add ubiquitinated signals to STING proteins using a two-step immunoprecipitation method. This suggests that TRIM56 may ubiquitinate a protein that can bind STING, rather than STING itself [64]. TRIM56 synthesizes ubiquitin chains that bind to NF-κB essential modifier (NEMO) and mediates the ubiquitination of NEMO to activate IKKβ, which is required for the activation of TBK1 and NF-κB [27,65] (Figure 2B). In the future, as technology advances, we believe that the relationship between TRIM56 and STING will eventually be determined.

#### 3.1.3. Enhancement of the Production of ISGs

IFN exerts its antiviral effect by inducing the expression of hundreds of ISGs [66]. After IFNα treatment, Kane et al. found that the overexpression of TRIM56 increased the expression of many ISGs. In this way, TRIM56 could suppress the expression of late HIV-1 genes, thereby establishing an anti-HIV status [67]. In addition, the overexpression of TRIM56 greatly enhanced extracellular the dsRNA-induced expression of IFN-β and ISGs, whereas the knockdown of TRIM56 severely impaired IRF3 activation, IFN-β and ISGs induction, the establishment of the antiviral state of TLR3 ligands, and severely impaired TLR3-mediated chemokine induction after hepatitis C virus (HCV) infection [24].

The known activity of ISGs is still insufficient to explain the antiviral effect of IFN, suggesting that more ISGs with antiviral activity need to be discovered. IFN-I itself enhances the expression of TRIM56 [22,48]. This suggests that TRIM56 is also a potential ISG. By inducing a positive feedback regulatory mechanism, TRIM56 plays an important role in the innate immune process.

### 3.2. TRIM56 Directly Targets Viruses

Viruses are a serious threat to the health of living organisms. TRIM56 can act as a direct antiviral restriction factor against many types of viruses, such as positive single-stranded RNA viruses, negative single-stranded RNA viruses, and DNA viruses (Table 1).

#### 3.2.1. Positive Single-Stranded RNA Viruses

The N-terminal protease (N(pro)) of bovine viral diarrhea virus (BVDV) is a proviral interferon antagonist capable of degrading interferon regulatory factor 3 (IRF3) via the proteasome. Although TRIM56 overexpression does not affect the protein levels of N(pro) and IRF3, it still interferes with BVDV replication. The anti-BVDV viral activity of TRIM56 is dependent on its E3 ubiquitin ligase activity and the integrity of its C-terminal region and is not due to a general enhancement of the interferon antiviral response [47].

TRIM56 does not improve cellular resistance to yellow fever virus (YFV), dengue virus serotype 2 (DENV2), or human coronavirus (HCoV) OC43. The anti-flavivirus (YFV, DENV2, and BVDV) function of TRIM56 requires the E3 ligase activity located in the N-terminal RING structural domain and the integrity of its C-terminal portion, whereas anti-HCoV-OC43 restriction only depends on TRIM56 E3 ligase activity. TRIM56 inhibits YFV, DENV2, and BVDV replication by impairing intracellular viral RNA replication, whereas it inhibits HCoV-OC43 progeny production later in the viral life cycle by targeting the viral packaging and release phase rather than intracellular viral RNA accumulation [68]. The above studies suggest that different structural domains of TRIM56 are adapted for different antiviral mechanisms.

Zika virus (ZIKV) infection is associated with microcephaly and other neurological disorders and is a serious threat to human health [69]. TRIM56 acts as an RNA-binding protein and binds to ZIKV RNA in infected cells. A recombinant TRIM56 fragment consisting of 392 C-terminal residues is able to directly bind ZIKV RNA in vitro. The overexpression of TRIM56, but not the E3 ligase-activating mutant or mutants lacking the short C-terminal portion, inhibits ZIKV RNA replication [46]. Thus, the C-terminus of TRIM56 interacts with ZIKV RNA, while the RING structural domain inhibits viral RNA replication.

Porcine epidemic diarrhea virus (PEDV) infection causes severe enteric disease in lactating piglets, resulting in significant economic losses to the swine industry [70]. TRIM56 expression levels were upregulated in cells infected with PEDV. The overexpression of TRIM56 increased the protein levels of TRAF3, a component of the TLR3 pathway, and upregulated IFN-β, ISG, and chemokine expression, which significantly activated downstream IRF3 and NF-κB signaling. The overexpression of TRIM56 inhibited PEDV replication, and the RING domain, N-terminal domain, or C-terminal portion of TRIM56 failed to inhibit PEDV replication [59].

Human immunodeficiency virus (HIV), also known as the AIDS virus, is a retrovirus that causes defects in the human immune system [71]. TRIM56 alters the release of HIV-1 [72]. TRIM56 enhances the induction of ISGs by IFNα and suppresses late HIV-1 gene expression [67].

TRIM56 exerts direct antiviral effects against several positive single-stranded RNA viruses, including members of the Coronaviridae family. 2019-nCoV belongs to the same family of coronaviruses as HCoV-OC43 and causes Coronavirus Disease 2019 (COVID-19). COVID-19 patients were found to have higher levels of TRIM56 expression. There was also a strong positive correlation between the expression levels of TRIM56 and VEGF [73]. This suggests that TRIM56 may have an anti-2019 nCoV function.

The antiviral activity of TRIM56 is virus-specific. TRIM56 was reported to be resistant to only seven positive single-stranded RNA viruses, including Flaviviridae YFV, DENV2, ZIKV, and BVDV; Retroviridae HIV-1; and OC43 and PEDV of the Coronaviridae family (Table 1). The overexpression of TRIM56 did not inhibit two positive single-stranded RNA viruses, encephalomyocarditis virus (EMCV) and HCV [47,68]. Whether TRIM56 affects other positive single-stranded RNA viruses remains to be investigated. Interestingly, the viral functions of anti-positive single-stranded RNA viruses are all dependent on the E3 ligase activity of TRIM56 [47,68].

#### 3.2.2. Negative Single-Stranded RNA Viruses

Therapeutic approaches for influenza remain very limited, and genetically mutated drug-resistant influenza virus strains often emerge [74]. Understanding novel virus–host interactions that alter influenza virus adaptations may reveal new targets/approaches for therapeutic intervention [75]. TRIM56 is able to specifically inhibit the RNA synthesis of influenza A and B viruses (Table 1). Interestingly, anti-influenza virus activity was not associated with E3 ligase activity, or B-box or coiled-coil structural domain. In contrast, the deletion of the 63-residue long C-terminal tail of TRIM56 abolished the antiviral function. In addition, the expression of this short C-terminal tail was as effective as full-length TRIM56 in inhibiting influenza virus replication [76]. 

The antiviral activity of TRIM56 is virus-specific. The overexpression of TRIM56 has been reported not to inhibit three negative single-stranded RNA viruses, VSV, Sendai virus, and human parapneumovirus [47,76]. Whether TRIM56 affects other negative single-stranded RNA viruses remains to be investigated.

#### 3.2.3. Double-Stranded DNA Viruses

TRIM56 expression is upregulated in IFN-treated HepG2 cells and Hepatitis B virus (HBV)-infected liver tissue. TRIM56 inhibits HBV replication with its RING and C-terminal structural domains. The C-terminal structural domain is essential for TRIM56 translocation from the cytoplasm to the nucleus during HBV infection (Table 1). TRIM56 ubiquitinates IκBα using the RING structural domain. This modification induces the phosphorylation of p65, which subsequently inhibits HBV core promoter activity, leading to the inhibition of HBV replication [77].

TRIM56 also promotes IFNα/β expression levels via the cGAS-STING signaling pathway and inhibits the replication of double-stranded DNA virus HSV-1 [25]. TRIM56-deficient mice show impaired production of IFNα/β and high susceptibility to lethal HSV-1 infection, but not to influenza A virus infection, because cGAS-STING-mediated immune responses are only directed against dsDNA and not against RNA viruses such as influenza A virus [25] (Table 1).

**Table 1:** Antiviral functions of TRIM56 against various viruses.

Virus	Genome	Mechanisms	Functions	Reference
BVDV	+ssRNA	RING and C-terminus	Inhibition of BVDV replication	[53]
YFV	+ssRNA	RING and C-terminus	Inhibition of YFV replication	[70]
DENV1/2	+ssRNA	RING and C-terminus	Inhibition of DENV1/2 replication	[52,70]
HCoV-OC43	+ssRNA	RING	Inhibition of packaging and release	[70]
ZIKV	+ssRNA	RING and C-terminus	Binding to ZIKV RNA and inhibition of ZIKV replication	[52]
PVDV	+ssRNA	RING, and N- and C- termini	Activation of TLR3 signaling and inhibition of PVDV replication	[64]
HIV-1	+ssRNA	-	Suppression of HIV-1 release	[69]
IAV, IBV	-ssRNA	C-terminus	Inhibition of IAV and IBV replication	[76]
HSV-1	dsDNA	-	Activation cGAS-STING signaling and inhibition of HSV-1 replication	[26]
HBV	dsDNA	RING	Ubiquitination of IκBα and inhibition of HBV replication	[50]

### 3.3. Other Pathogens

The hallmark of Salmonella typhi infection is an acute intestinal inflammatory response, which is mediated by the action of secreted bacterial effector proteins [78]. Inflammation-promoting Salmonella effector SopA is an E3 ligase similar to HECT [79,80]. By targeting TRIM56 and TRIM65, SopA can stimulate innate immune signaling with two innate immune receptors, RIG-I and MDA5, respectively [69]. However, Fiskin et al. proposed the opposite mechanism. They found that endogenous TRIM56 and TRIM65 protein levels decreased under standard Salmonella infection conditions. SopA inhibited TRIM56 E3 ligase activity by occluding the E2 binding surface of TRIM56. At the same time, SopA ubiquitinates TRIM56, leading to proteasomal degradation during infection [81]. Whether TRIM56 plays a role in other types of bacterial infections remains to be investigated.

## 4. The Function of TRIM56 in Tumors

**Figure 3:**
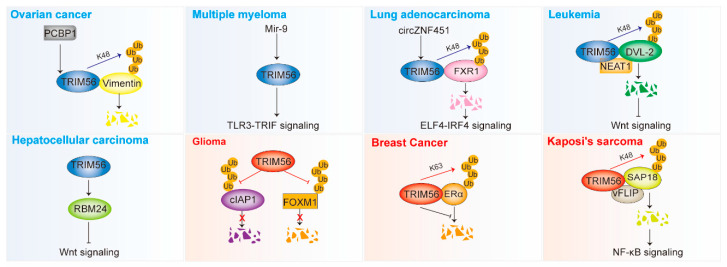
TRIM56 is involved in the development of cancer (see also Table 2). TRIM56 plays a dual role in tumors. The red color in the figure represents the cancer-promoting function and the blue color represents the cancer-suppressing function. TRIM56 promotes the development of glioma, breast cancer, and Kaposi’s sarcoma, but is an oncogenic repressor in ovarian cancer, multiple myeloma, lung adenocarcinoma, hepatocellular carcinoma, and leukemia. TRIM56 affects multiple signaling pathways, including the TLR3-TRIF pathway, the ELF4-IRF4 pathway, the Wnt pathway, and the NF-κB pathway. TRIM56 mediates the ubiquitin degradation of key proteins, such as Vimentin, FXR1, DVL2, and SAP18. TRIM56 can stabilize some key proteins via K63 ubiquitination (ERα) and deubiquitination (cIAP1 and FOXM1). Ub, ubiquitin.

By regulating various signaling pathways and proteins in an E3 ligase-dependent or -independent manner, TRIM56 plays different roles in different tumors. It inhibits ovarian cancer, multiple myeloma, lung adenocarcinoma, hepatocellular carcinoma, and leukemia; however, it promotes the development of glioma, breast cancer, and Kaposi’s sarcoma (Figure 3 and Table 2).

### 4.1. Tumor Suppression

#### 4.1.1. Ovarian Cancer

Ovarian cancer is a gynecologic oncologic disease and one of the major female lethal cancers [82]. Epithelial-to-mesenchymal transition (EMT) leads to tumor metastasis, which accelerates tumor progression [83]. Vimentin is an important protein that regulates EMT and cancer progression in ovarian cancer [84]. TRIM56 is able to ubiquitinate and downregulate Vimentin. The TRIM56 inhibition of ovarian cancer migration and invasion in vitro occurs via an inhibitory effect on Vimentin [33]. TRIM56 expression is post-transcriptionally regulated at the translational level by RNA-binding protein poly r(c)-binding protein 1 (PCBP1) [85]. PCBP1 promotes ovarian cancer migration and invasion in vitro by inhibiting TRIM56 translation, reducing its protein levels, thereby inducing Vimentin expression [33,85].

#### 4.1.2. Multiple Myeloma

Multiple myeloma (MM) is a group of plasma cell malignancies characterized by the extensive clonal proliferation of tumor plasma cells in the bone marrow [86]. MM accounts for approximately 10% of hematologic neoplastic diseases [87]. The bone marrow microenvironment and cytokines such as interleukin (IL)-6 and TNF (tumor necrosis factor)-α play an important role in the growth and survival of MM cells and are associated with the clinical presentation and prognosis of MM [88]. The expression of TRIM56 is significantly decreased in MM cells. TRIM56 inhibits cell proliferation and produces inflammatory cytokines by activating the TLR3/TRIF signaling pathway [34]. Huang et al. found that cell lines from early MM patients showed upregulated miR-9 expression, which promoted MM cell proliferation and reduced apoptosis. TRIM56 is a target protein of miR-9 that reverses miR-9-mediated proliferation and anti-apoptotic effects. Thus, miR-9 promotes MM development and progression with the regulation of the TRIM56/NF-κB pathway [89].

#### 4.1.3. Lung Cancer

Lung cancer is the most common and lethal malignancy, with lung adenocarcinoma accounting for up to 40% of cases [90]. The reduced expression of TRIM56 in lung adenocarcinoma is associated with poor prognosis. The overexpression of TRIM56 inhibits the invasion and migration of lung adenocarcinoma cells [35]. In the treatment of advanced lung cancer, immunotherapy has achieved some success, but the problem of immunotherapy resistance cannot be ignored [91,92]. Exosomal circZNF451 was upregulated in patients with progressive disease compared with lung adenocarcinoma patients in partial remission after PD1 blockade therapy and was associated with a poor clinical prognosis. Exosomal circZNF451 was able to target RNA-binding protein FXR1 in macrophages and promote the ubiquitination of FXR1 via the E3 ubiquitin ligase TRIM56, which in turn activated the ELF4-IRF4 pathway, leading to M2 polarization and suppressive immune microenvironment in macrophages. Exosomal circZNF451 inhibits anti-PD1 therapy in lung adenocarcinoma by polarizing macrophages in complex with TRIM56 and FXR1 [93]. Thus, TRIM56 may serve as a potential therapeutic target and a novel predictive marker for PD1 inhibitor resistance in lung cancer.

#### 4.1.4. Leukemia

DVL2 is a key regulator of Wnt signaling, which stabilizes β-catenin by catabolizing the APC/Axin/CK1α/GSK3β degradation complex [94]. DVL2 expression levels are closely correlated with Wnt activity and tumor progression [31,95]. The TRIM56-mediated degradation of DVL2 inactivates Wnt signaling and thus inhibits tumor development. Nuclear paraspeckle assembly transcript 1 (NEAT1) localizes to the nucleus and is able to inhibit AML stem cell self-renewal and leukemogenesis by activating Wnt signaling [36]. Alternative splicing (AS) often alters the function of proteins, which in turn affects tumor development [96]. However, heterodimer NEAT1 is localized in the cytoplasm and is able to interact with TRIM56 and DVL2 by enhancing TRIM56-mediated DVL2 degradation, thereby inactivating Wnt signaling [36]. Targeting DVL2 using TRIM56- or DVL2-interacting NEAT1 truncators may be a potential strategy for the treatment of AML.

#### 4.1.5. Hepatocellular Carcinoma

Yang et al. found that downregulated TRIM56 in hepatocellular carcinoma (HCC) patient samples was strongly associated with pathological stage and prognosis [37]. TRIM56 negatively regulated key genes in Wnt signaling, β-catenin, c-Myc, RBM24, MMP-9, and cyclin D1, as well as Wnt. Among them, RBM24 was shown to be a downstream target gene of TRIM56. The overexpression of TRIM56 inhibited cell proliferation, whereas the knockdown of TRIM56 had the opposite effect. TRIM56 inhibited HCC proliferation by inactivating Wnt signaling and targeting RBM24 [37].

Hepatocellular carcinoma has been associated with viral infections of type B and C [97]. TRIM56 can inhibit the replication of HBV [77]. In addition, TRIM56 was able to promote the induction of TLR3-mediated chemokines after HCV infection [24].

### 4.2. Tumor Promotion

#### 4.2.1. Glioma

TRIM56 expression is significantly increased in glioblastoma tissues and cell lines. High TRIM56 expression is associated with a poor prognosis in glioma patients [29,30]. TRIM56 can downregulate the ubiquitination level of cIAP1, thereby reducing the degradation of cIAP1 [30]. cIAP1 belongs to the inhibitors of apoptosis (IAP) family, which regulates the cell cycle and tumor development [98]. Several studies have shown that cIAP1 is highly expressed in various human cancers and plays a key oncogenic role [98,99]. In glioma, TRIM56 does not function as an E3 ligase but as a deubiquitinating enzyme to stabilize the expression of apoptosis inhibitor cIAP1, thereby promoting glioma progression [30]. Recurrent glioblastoma is characterized by resistance to radiotherapy or chemotherapy. TRIM56 increases FOXM1 protein levels and enhances FOXM1 by means of deubiquitination. TRIM56 inhibits the radiosensitivity of human glioblastoma by regulating FOXM1-mediated DNA repair. Targeting TRIM56 may be an effective approach to reverse radioresistance in glioblastoma recurrence [29]. Interestingly, TRIM56 in gliomas function as deubiquitinating enzymes rather than E3 ligases.

#### 4.2.2. Breast Cancer

Breast cancer is the most common cancer in women worldwide [100,101]. The knockdown of TRIM56 enhances the proliferation and metastasis of breast cancer cells. The expression of TRIM56 is positively correlated with ERα and PR in breast cancer samples and is associated with poor prognosis in patients treated with endocrine therapy. Approximately 60–70% of breast cancer patients are Erα-positive [102]. Estrogen-selective modulators, such as tamoxifen, are emerging as effective agents for controlling ERα breast cancer progression [103]. However, tamoxifen resistance develops during long-term treatment and cancer progression [104]. TRIM56 catalyzes the formation of K63-linked polyubiquitin chains of ERα, thereby prolonging the stability of the ERα protein [31]. Breast cancer proliferation requires transduction via the ERα signaling pathway. Therefore, TRIM56-targeted therapy may address treatment resistance, thereby inhibiting cancer cell proliferation.

#### 4.2.3. Kaposi’s Sarcoma

Kaposi’s Sarcoma (KS) is a common AIDS-associated cancer caused by KS-associated herpesvirus (KSHV) infection [105]. KSHV encodes viral FLICE inhibitory protein (vFLIP), a viral oncogenic protein. vFLIP promotes cell migration, invasion, and angiogenesis by downregulating the SAP18-HDAC1 complex. Specifically, vFLIP degrades SAP18 via the ubiquitin–proteasome pathway by recruiting E3 ubiquitin ligase TRIM56, which ultimately activates the NF-κB signaling pathway [32]. Interestingly, KSHV is closely associated with the development of KS, primary exudative lymphoma (PEL), and other diseases [106]. Moreover, the deletion of the TRIM56 gene has been found in PEL patients [107]. The relationship between TRIM56 and KSHV, and KSHV-related tumors needs to be further investigated.

### 4.3. Regulation of TRIM56 Expression in Tumors

**Figure 4:**
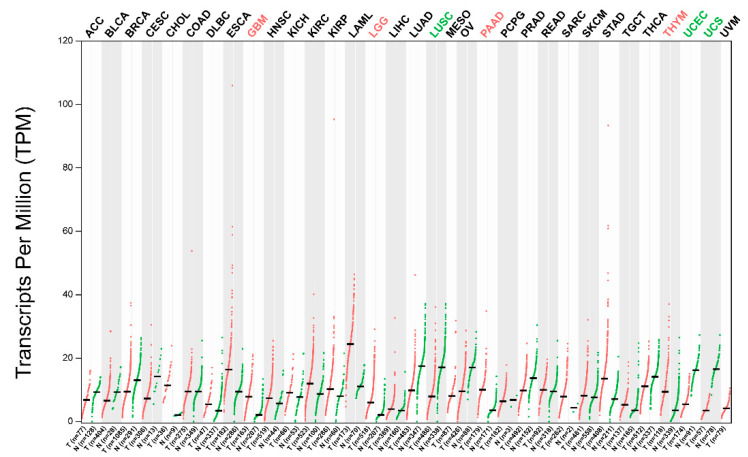
TRIM56 is aberrantly expressed in several types of tumors. By analyzing the data in the TCGA database, we found that the expression levels of TRIM56 were significantly low in lung squamous cell carcinoma (LUSC), uterine corpus endometrial carcinoma (UCEC), and uterine carcinosarcoma (UCS), and significantly high in pancreatic adenocarcinoma (PAAD), glioblastoma (GBM), lower-grade glioma (LGG), and thymoma (THYM). Data were analyzed using GEPIA 2.0.

TRIM56 is aberrantly expressed in a variety of tumors. TRIM56 was lowly expressed in multiple myeloma [88], ovarian cancer [85], lung adenocarcinoma [35], and hepatocellular carcinoma [37]. TRIM56 was highly expressed in glioma [29,30]. Furthermore, by analyzing the data in the TCGA database, we found that the expression levels of TRIM56 were significantly low in lung squamous cell carcinoma, uterine corpus endometrial carcinoma, and uterine carcinosarcoma, and significantly high in pancreatic adenocarcinoma, glioblastoma, lower-grade glioma, and thymoma [108] (Figure 4). In addition to the above tumors, TRIM56 was highly expressed in living patients with muscle-invasive bladder cancer (MIBC) [109]. The function and regulatory mechanisms of TRIM56 in the above tumors remain to be investigated. In addition, the upstream regulatory mechanisms of TRIM56 are not well understood. In ovarian cancer, PCBP1 inhibits TRIM56 translation [85]. In multiple myeloma, mir-9 downregulates TRIM56 expression [89]. In lung adenocarcinoma, mir-542 and mir-627 have the potential to inhibit TRIM56 expression [35].

## 5. Concluding Remarks and Future Perspectives

This review summarizes the role of TRIM56 in antiviral processes and the development of tumorigenesis. Elucidating the altered expression of TRIM56 and its potential mechanisms in the pathophysiology of cancer and other diseases may provide insights for the development of new and more effective therapeutic strategies. However, there are still no reports on the clinical applications of TRIM56 in small-molecule therapy. A further understanding of the crystal structure of TRIM56 and its ligand-binding complexes could refine the structure-based design for the development of specific small molecules targeting TRIM56, ultimately leading to therapeutic applications.

As an E3 ubiquitin ligase, TRIM56 catalyzes the ubiquitination modification of substrates [23,26,31,32,33,36,61,77]. The fate of the substrate protein depends on the lysine used to form the ubiquitin molecule of the heteropeptide bond. Different ubiquitination chain lengths (monoubiquitination and polyubiquitination) and a wide variety of ubiquitination chain types (linked by Met1, Lys6, Lys11, Lys27, Lys29, Lys33, Lys48, and Lys63) play an extremely important role in protein activity, protein–protein interactions, and protein subcellular localization [39,110]. TRIM56 is able to catalyze the formation of K48, K63-linked, or M1-linked ubiquitination [111]. In addition, biological roles of TRIM56 independent of E3 ligases have been identified, including RNA binding and deubiquitinating enzyme activity [29,30,46].

The innate immune response is the first line of host defense and is characterized by the production of IFN-I and ISGs to limit viral infection and transmission [112,113,114]. Many studies have shown that TRIM56 plays a key role in the precise coordination of key signaling molecules and their associated pathways. Here, we discuss the current mechanisms regarding the involvement of TRIM56 in the regulation of TLRs, the cGAS-STING pathway, and downstream ISGs [23,26,61]. Whether TRIM56 regulates the RLRs pathway remains to be further investigated. In addition, TRIM56 is able to regulate innate antiviral signaling in a ubiquitination-independent manner, and the specific mechanisms of regulation remain to be explored.

The C-terminal region of TRIM56 mediates protein–protein or protein–RNA interactions between TRIM56 and cellular viral proteins/RNAs and can inhibit viral RNA replication. In addition, the E3 ligase activity of TRIM56 may regulate post-translational modifications of viral proteins and/or host factors to inhibit the replication of positive-stranded RNA viruses. Although TRIM56 is widely expressed in many tissues, the highest expression levels of the protein were detected in the lung and stomach [47]. The presence of pathogenic microorganisms in the respiratory and gastrointestinal tracts, which are continuously exposed to the external environment, may account for the differences in tissue distribution [115].

TRIM56 has been reported to exert oncogenic or tumorigenic potential in solid tumors and hematological cancers [116,117]. The importance of exploring the function of TRIM56 in various malignancies comes not only from the understanding of the key mechanisms of tumor development but also from the important translational potential. In recent years, TRIM family proteins have made some progress in targeted cancer therapy, such as TRIM8-targeted approaches for chemotherapy-resistant colorectal cancer and TRIM24-targeted regimens for glioblastoma [118,119]. TRIM56 can affect tumor cell proliferation, apoptosis, and metastasis by regulating downstream molecules [29,30,31,32,33,34,35,36,37]. However, the effect of TRIM56 on tumor immunity is still unknown. TRIM56 can modulate the innate immune response and promote the production of type I IFNs and ISGs [23,24,25,26,27,65]. Notably, the innate immune response plays an important role in cancer immune escape [120,121]. Therefore, exploring the effect of TRIM56 on tumor immune response is a future research direction.

## Figures and Tables

**Table 2:** Expression and clinical significance of TRIM56 in various cancers.

Cancer Type	Expression	Mechanisms	Functions	References
Ovarian cancer	-	Ubiquitination and downregulation of Vimentin	Inhibition of migration and invasion	[35]
Multiple myeloma	Decrease	Production of inflammatory cytokines with activation of the TLR3/TRIF signaling pathway	Inhibition of cell proliferation	[36]
Lung cancer	Decrease	-	Inhibition of the invasion and migration of lung adenocarcinoma cells	[37]
Leukemia	-	Ubiquitination and downregulation of DVL2	Inhibition of AML stem cell self-renewal and leukemogenesis	[38]
Hepatocellular carcinoma	Decrease	Inactivation of Wnt signaling and targeting of RBM24	Inhibition of HCC proliferation	[39]
Glioma	Increase	Deubiquitination of and increase in FOXM1 and cIAP1 protein levels	Promotion of glioma progression and inhibition of radiosensitivity of glioblastoma	[31,32]
Breast cancer	-	Ubiquitination of and increase in the stability of ERα	Promotion of proliferation	[33]
Kaposi’s sarcoma	-	Ubiquitination and downregulation of SAP18	Promotion of cell invasion and angiogenesis	[34]

## Data Availability

All data relevant to this review are included in the text, references, table, and figures.

## Abbreviations

TRIM	Tripartite-motif
RING	Really interesting new gene
RBCC	RING-B-box-Coiled-Coil
FNIII	Fibronectin type III repeat region
ACID	Acid-rich region
MATH	Meprin and TRAF-homologous structural domain
ARF	ADP-ribosylation factor family structural domain
FIL	Filamine-type IG structural domain
BROMO	Bromodomain
TM	Transmembrane region
IFN	Interferon
TLRs	Toll-like receptors
cGAS	Cyclic GMP-AMP synthase
STING	Stimulator interferon genes HECT: homologous to E6AP C-terminus
DVL2	Dishevelled-2
SAP18	Sin3A associated protein 18
TAK1	TGF-β-activated kinase 1
PAMPs	Pathogen-associated molecular patterns
PRRs	Pattern recognition receptors
RIG-I	Retinoic-acid inducible gene-I
RLRs	RIG-I-like receptors
ISGs	Interferon-stimulated genes
IFN-I	Type I IFN
MyD88	Myeloid differentiation factor 88
IRAK1/4	IL-1 receptor-associated kinase 1/4
TRAF6	TNF receptor-associated factor 6
TAB2	TAK1/MAP3K7 binding protein 2
IκKα/β/γ	IκB kinase α/β/γ
TRIF	Toll-IL-1 receptor (TIR) domain-containing adaptor inducing IFN-β
cGAMP	Cyclic GMP-AMP
ER	Endoplasmic reticulum
HSV-1	Herpes simplex virus-1
UBXN3B	Ubiquitin regulatory X domain-containing proteins 3B
VSV	Vesicular stomatitis virus
NEMO	NF-κB essential modifier
HCV	Hepatitis C virus
N(pro)	N-terminal protease
BVDV	Bovine viral diarrhea virus
IRF3	Interferon regulatory factor 3
YFV	Yellow fever virus
DENV2	Dengue virus serotype 2
HCoV	Human coronavirus
ZIKV	Zika virus
PEDV	Porcine epidemic diarrhea virus
HIV	Human immunodeficiency virus
COVID-19	Coronavirus Disease 2019
EMCV	Encephalomyocarditis virus
HBV	Hepatitis B virus
EMT	Epithelial-to-mesenchymal transition
PCBP1	Poly r(c)-binding protein 1
MM	Multiple myeloma
IL	Interleukin
TNF	Tumor necrosis factor
NEAT1	Nuclear paraspeckle assembly transcript 1
HCC	Hepatocellular carcinoma
IAPs	Inhibitors of apoptosis
KS	Kaposi’s sarcoma
KSHV	KS-associated herpesvirus
vFLIP	Viral FLICE inhibitory protein
PEL	Primary exudative lymphoma
MIBC	Muscle-invasive bladder cancer
